# Batch correction of microarray data substantially improves the identification of genes differentially expressed in Rheumatoid Arthritis and Osteoarthritis

**DOI:** 10.1186/1755-8794-5-23

**Published:** 2012-06-08

**Authors:** Peter Kupfer, Reinhard Guthke, Dirk Pohlers, Rene Huber, Dirk Koczan, Raimund W Kinne

**Affiliations:** 1 Research Group Systems Biology/Bioinformatics, Leibniz Institute for Natural Product Research and Infection Biology – Hans Knöll Institute, Jena, Germany; 2 Experimental Rheumatology Unit, Department of Orthopedics, University Hospital Jena, Friedrich Schiller University, Jena; 3 Present address: Center of diagnostics GmbH, Chemnitz Hospital, Chemnitz, Germany; 4 Present address: Institute of Clinical Chemistry, Hannover Medical School, Hannover, Germany; 5 Institute of Immunology, University of Rostock, Rostock, Germany

**Keywords:** Microarray analysis, Batch correction, Rheumatoid arthritis, Osteoarthritis, Collagen, Extracellular matrix

## Abstract

**Background:**

Batch effects due to sample preparation or array variation (type, charge, and/or platform) may influence the results of microarray experiments and thus mask and/or confound true biological differences. Of the published approaches for batch correction, the algorithm “Combating Batch Effects When Combining Batches of Gene Expression Microarray Data” (*ComBat*) appears to be most suitable for small sample sizes and multiple batches.

**Methods:**

Synovial fibroblasts (SFB; purity > 98%) were obtained from rheumatoid arthritis (RA) and osteoarthritis (OA) patients (n = 6 each) and stimulated with TNF-α or TGF-β1 for 0, 1, 2, 4, or 12 hours. Gene expression was analyzed using Affymetrix Human Genome U133 Plus 2.0 chips, an alternative chip definition file, and normalization by Robust Multi-Array Analysis (*RMA*). Data were batch-corrected for different acquiry dates using *ComBat* and the efficacy of the correction was validated using hierarchical clustering.

**Results:**

In contrast to the hierarchical clustering dendrogram before batch correction, in which RA and OA patients clustered randomly, batch correction led to a clear separation of RA and OA. Strikingly, this applied not only to the 0 hour time point (i.e., before stimulation with TNF-α/TGF-β1), but also to all time points following stimulation except for the late 12 hour time point. Batch-corrected data then allowed the identification of differentially expressed genes discriminating between RA and OA. Batch correction only marginally modified the original data, as demonstrated by preservation of the main Gene Ontology (GO) categories of interest, and by minimally changed mean expression levels (maximal change 4.087%) or variances for all genes of interest. Eight genes from the GO category “extracellular matrix structural constituent” (5 different collagens, biglycan, and tubulointerstitial nephritis antigen-like 1) were differentially expressed between RA and OA (RA > OA), both constitutively at time point 0, and at all time points following stimulation with either TNF-α or TGF-β1.

**Conclusion:**

Batch correction appears to be an extremely valuable tool to eliminate non-biological batch effects, and allows the identification of genes discriminating between different joint diseases. RA-SFB show an upregulated expression of extracellular matrix components, both constitutively following isolation from the synovial membrane and upon stimulation with disease-relevant cytokines or growth factors, suggesting an “imprinted” alteration of their phenotype.

## Background

Gene expression microarray technology measures the expression of thousands of genes in one single array by using multiple probes to assay each transcript. It is a widely-used tool for identifying genes whose expression changes in response to specific treatments. There are several concerns with regard to the reliability of DNA microarray technology in the study of diseases.

Microarray experiments are costly and time-consuming. Many studies use multiple arrays, with experiments performed at different times, on different array charges or even with different microarray platforms. The resulting gene expression data could thus be affected by non-biological variables. These systematic differences between the measurements are commonly referred to as “batch effects”. There are several causes for batch effects, as outlined below:

 • Ambient conditions during the sample preparation and handling, such as room temperature and ozone levels

 • Storage and shipment conditions of the biological samples and/or the arrays

cRNA/cDNA synthesis

 • Amplification, labeling, and hybridization protocol: Use of reagents from different batches

 • Sites/laboratories: Different laboratories have different operating procedures

 • Chip type/charge/platform: The array quality varies from batch to batch

 • Washing conditions: Temperature, ionic strength

 • Scanner: Type, settings, and the calibration drift [1].

Although some of these batch effects can be minimized or even prevented with appropriate precautions and experimental design, certain batch effects are unavoidable. Some studies require large sample sizes and have to be carried out over many months or sometimes years. In clinical experiments, experiments are often driven by the availability of the samples, which cannot be specifically controlled for in the original study design. Combining data from different batches without removing batch effects can give rise to misleading results, since the bias introduced by the non-biological nature of the batch effects can be strong enough to mask and/or confound true biological differences. Thus, there is a need to identify and remove these masking effects before further processing.

Microarray signal intensity normalization has been widely used to adjust for experimental artifacts between the samples. The effect of the normalization is to increase the precision of multi-array measurements through calibration of the signal intensity distributions. There are several methods for normalization including *MAS5*, Robust Multi-Array Analysis (*RMA*), and *dChip* for Affymetrix chips, median scaling for *GE-CodeLink* microarrays, and LOWLESS-based methods for cDNA two-color microarrays. Common to all normalization methods is that they are not specifically designed to remove batch effects reflected by systematic differences between two or more groups of samples. Consequently, batch effects may often remain after normalization. However, of thousands of papers dealing with DNA microarrays published in the last 5 years (>32,000), only few address the potential existence of batch effects and/or their correction. Of the 219 papers using microarray data published from January 1 to July 1, 2010, not even ten percent took this issue into account (NCBI GEO database, studies with more than 30 samples) [2].

There are several published approaches to identify and remove batch effects [1,3]. An Empirical Bayes method called “Combating Batch Effects When Combining Batches of Gene Expression Microarray Data” (*ComBat*) estimates parameters for location and scale adjustment of each batch for each gene independently [4-6]. In the present study, this method was applied to a data subset of 24 arrays (of a total of 120) in order to remove batch effects due to different acquiry dates of the microarray analyses. In contrast to the hierarchical clustering dendrogram before batch correction, in which rheumatoid arthritis (RA) and osteoarthritis (OA) patients clustered randomly, batch correction led to a clear separation of the RA and OA groups. Batch-corrected data then allowed an unequivocal identification of differentially expressed genes discriminating between RA and OA patients.

## Materials and methods

### Patients

Synovial membrane samples were obtained following tissue excision upon joint replacement/synovectomy from RA and OA patients (n = 6 each; all Caucasian; Tables 1 and 2) at the Clinic of Orthopedics, Waldkrankenhaus “Rudolf Elle” (Eisenberg, Germany). Informed patient consent was obtained and the study was approved by the ethics committee of the University Hospital Jena. RA patients were classified according to the American College of Rheumatology (ACR) criteria valid in the sample assessment period [7], OA patients according to the respective criteria for osteoarthritis [8]. Negative purification of primary synovial fibroblasts (SFB) from RA and OA patients (purity: > 98%) was performed as previously described [9].

**Table 1:** Clinical data of patients

**Sample name**	**Gender/ Age**	**Disease duration**	**RF**	**ESR (mm/h)**	**CRP (mg/ml)**	**ARA criteria**	**Concurrent medication**
**Rheumatoid Arthritis**							
EB87	F/65	12	+	50,00	106,7	5	NSAIDs
EB88	F/62	10	+	90,00	169,5	6	NSAIDs
EB213	F/69	15	+	94,00	99,1	4	NSAIDs, Leflunomide, Prednis
EB220	F/57	20	+	23,00	2,3	4	NSAID, MTX, Prednis.
EB221	F/66	12	+	7,00	5,4	4	Lantarel, MTX
EB227	F/49	25	+	12,00	2,4	5	NSAIDs, MTX, Prednis.
**Osteoarthritis**							
EB168	F/68	20	-	18,00	20,3	0	NSAIDs
EB173	F/81	2,5	-	38,00	32,0	2	NSAIDs, Prednis.
EB190	M/56	5	-	6,00	0,5	1	NSAIDs
EB194	F/79	8	-	28,00	0,3	1	NSAIDs
EB202	M/65	5	-	7,00	3,0	0	NSAIDs
EB205	F/55	3	-	47,00	36,0	0	NSAIDs

ARA, American Rheumatism Association (now American College of Rheumatology); CRP, C-reactive protein (normal range <5 mg/l); ESR, erythrocyte sedimentation rate; F, female; M, male; MTX, methotrexate; NSAIDs, non steroidal anti-inflammatory drugs; RF, rheumatoid factor;-,negative; +, positive.

**Table 2:** Sample features (incl. acquiry date) and clinical diagnosis of RA and OA patients

**Batch**	**Acquiry date**	**Sample ID**	**Treatment**	**Diagnosis**
1	2006-04-07	EB87.TGF.0 h	TGF-β1	RA
		EB87.TNF.0 h	TNF-α	
2	2006-11-30	EB190.TGF.0 h	TGF-β1	OA
		EB190.TNF.0 h	TNF-α	
3	2006-12-07	EB202.TGF.0 h	TGF-β1	
		EB202.TNF.0 h	TNF-α	
		EB205.TGF.0 h	TGF-β1	
		EB205.TNF.0 h	TNF-α	
4	2006-12-12	EB220.TGF.0 h	TGF-β1	RA
		EB220.TNF.0 h	TNF-α	
		EB221.TGF.0 h	TGF-β1	
		EB221.TNF.0 h	TNF-α	
5	2009-03-04	EB173.TGF.0 h	TGF-β1	OA
		EB173.TNF.0 h	TNF-α	
		EB227.TGF.0 h	TGF-β1	RA
		EB227.TNF.0 h	TNF-α	
6	2009-03-14	EB194.TGF.0 h	TGF-β1	OA
		EB194.TNF.0 h	TNF-α	
		EB213.TGF.0 h	TGF-β1	RA
		EB213.TNF.0 h	TNF-α	
7	2009-03-26	EB88.TGF.0 h	TGF-β1	
		EB88.TNF.0 h	TNF-α	
		EB168.TGF.0 h	TGF-β1	OA
		EB168.TNF.0 h	TNF-α	

All samples (total 120 arrays) were derived from 5 different time points of the respective stimulation experiment. Batch = Integer label for the Batch Correction; Acquiry date = date of hybridization of the microarray; Sample ID = unique array identification; Stimulation = Stimulation of the samples; Diagnosis = Disease of the patient.

### Cell stimulation and isolation of total RNA

At the end of the fourth passage, the SFB were washed in serum-free DMEM and then stimulated by 10 ng/ml TNF-α or TGF-β1 (PeproTech, Hamburg, Germany) in serum-free DMEM for 0, 1, 2, 4, or 12 hours. At each time point, the medium was removed and the cells were harvested after treatment with trypsin (0.25% in versene; Invitrogen, Karlsruhe, Germany). After washing with phosphate-buffered saline, they were lysed with RLT buffer (Qiagen, Hilden, Germany) and frozen at −70°C. Total RNA was isolated using the RNeasy Kit (Qiagen) according to the supplier's recommendation.

### Microarray analysis

Analysis of gene expression was performed using U133 Plus 2.0 RNA microarrays (Affymetrix, Santa Clara, CA, USA; total of 120 microarrays). Labeling of RNA probes, hybridization, and washing were carried out according to the supplier's instructions. Microarrays were analyzed by laser scanning (Hewlett-Packard Gene Scanner). Original data from microarray analysis are accessible through Gene Expression Omnibus series accession number GSE13837 [10].

For annotating the genes, the alternative Chip Definition File (CDF) of Ferrari *et al.* was used to resolve the problem of choosing reliable and non-contradictory probesets for each transcript [11]. Several publications demonstrated the advantage of such alternative CDFs for the removal of cross-hybridization and other system-based biases.

The microarray data were preprocessed using *RMA* in the default configuration for background adjustment and normalization.

### ComBat

For Batch correction of the patient data (Table 2), the Empirical Bayes' (EB) method *ComBat* was used (non-parametric prior method) [5]. EB methods are very appealing in microarray analyses because of their ability to robustly handle high-dimensional data derived from small sample sizes. EB methods are primarily designed to “borrow information” from a certain number of genes and/or experimental conditions in order to obtain better estimates or more stable inferences for the expression of all genes. In several papers, EB methods were designed to stabilize the expression values/ratios for genes with extreme values or else the variance of genes or gene groups by shrinking variances across all other genes, possibly diminishing the effects of artifacts in the data [6,12-19]. Johnson *et al.* extended the EB methods to the problem of adjusting for batch effects in microarray data, which are not addressed by the use of one or several normalization procedures [5]. Johnson *et al.* published a location and scale (L/S) adjustment method for batch correction, which is available as R-package *ComBat* at the developer's homepage [20]. In contrast to other L/S methods, this method is the only procedure currently known to robustly adjust batches with small sample sizes.

As other L/S adjustments, *ComBat* assumes that the batch effects can be modeled by standardizing means and variances across batches. It uses a straightforward L/S adjustment to independently center the mean and standardize the variance for each gene in each batch. This method incorporates systematic batch biases common across several genes to make adjustments on the assumption that phenomena resulting in batch effects often affect many genes in a similar way (i.e., increased expression, higher variability, etc.). To determine the data parameters which describe the particular L/S model [5], *ComBat* estimates the L/S model parameters that best represent the batch effects by “pooling information” across some or all genes in each batch in order to “shrink” the parameter estimates and thereby reduce the influence of batch effects.

In the present study, a modified method of *ComBat* was used to correct for batch effects among arrays generated at different dates. The algorithm was modified in order to allow processing of *RMA*-normalized data instead of *dChip*-normalized data. The Sample Information File was created as described in the *ComBat* manual. The creation date was tagged as “batch effect” and the parameters time point (total of 5), disease group (RA and OA), and stimulation (TNF-α and TGF-β1) were marked as covariates for every array.

### Limma

To identify differentially expressed genes, the software *Limma* was used. *Limma* is an R-package for differential expression analysis of data arising from microarray experiments [21]. The package is designed to analyze complex experiments involving simultaneous comparisons between many RNA targets. The basic concept is to fit a linear model to the expression data for each gene. The expression data from experiments are represented as a data matrix with rows corresponding to probe sets and columns to arrays. For the analysis with linear models, the approach requires two matrices. First, the design matrix was created, which provides a representation of the different RNA targets. Secondly, the contrast matrix was generated, which allows to assign the coefficients defined by the design matrix to contrasts of interests (in our case time point 0 hours, RA versus OA patients, Table 2). Afterwards, the linear model was fitted using the *lmFit* function, which fully models the systematic part of the data. The actual analysis was analogous to the classic *t*-test except that the *eBayes*-function was used which employs the shrunken empirical Bayes estimates [21]. Differentially expressed genes (DEG) were obtained by using the *toptable* function and user-specific thresholds (i.e., 2-fold changes in gene expression and a q-value with a threshold of 0.05; [22]). The q-value (FDR adjusted p-value) was calculated using the Benjamini and Hochberg’s method [21].

### GOstats

The Bioconductor package *GOstats* shows substantial improvements for testing the association between Gene Ontology (GO) terms and a given gene list [23]. Falcon *et al.* implemented a conditional hypergeometric (*hg*) test that uses the relationships among GO terms to address the hierarchical structure of GO. To use this *hg* test, the gene universe (in this case containing all genes on the microarray) and a list of selected genes from that universe was defined (DEG). After setting a cutoff for the adjusted p-value of the *hg* test, *GOstats* returns a *GOHyperGResult* with a summary of the test performed and the number of significant GO terms. Furthermore, the *GOHyperGResult* contains the expected gene count and actual gene count for each GO term.

### GPower

The Tool GPower 3.1.3 was used for post-hoc calculation of the power (1- ß error probability) of the *t*-test with the error probability alpha = 0.05, where the sample sizes of the two groups (RA, OA) were 12 each and the respective mean values and SD of the two groups were derived from Additional file 1: Table S1 [24,25].

## Results

### Clustering of RA and OA patients before and after batch correction

Starting with *RMA*-normalized data of the 24 arrays for time point 0, standard hierarchical clustering dendrograms (generated using the R function *hclust* with euclidean distances) were employed to monitor the effect of the batch correction by *ComBat*. These dendrograms measure inter-cluster distances between the arrays. The resulting distances can be interpreted as the dissimilarity of the arrays

**Figure 1:**
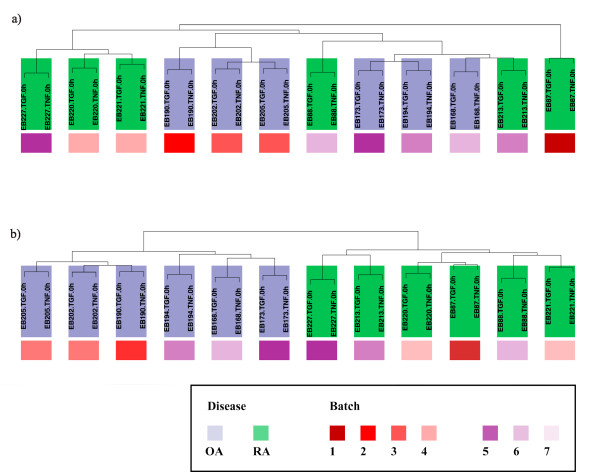
**Hierarchical clustering of uncorrected and batch-corrected data from time point 0 hours: a) The uncorrected data form clusters reflecting the 7 different acquiry dates (red shades for arrays generated in 2006; blue shades for those generated in 2009; for precise definition of the individual acquiry dates see Table **2**).** In contrast, RA and OA are not grouped. **b**) The *ComBat*-corrected data (7 batches) form clusters reflecting the diseases (RA and OA) instead of the acquiry dates.

Before batch correction, the normalized data formed clear clusters for the different batches (7 different acquiry dates for groups of 2 – 4 individual Affymetrix chips; Figure 1a). Except for 2 patients (extreme left and extreme right), two main clusters were formed, separating the microarrays created in 2006 (red shades) from those generated in 2009 (blue shades). Indeed, the company providing the hybridization agents for the microarrays confirmed a change in the chemical components used in the production process in the year 2007. As a consequence of the batch effect, several unique clusters were observed for the RA and OA patients (right and left branches of the dendrogram), but other clusters were mixed and contained members of both groups. In addition, patient EB87 (extreme right cluster) showed the highest dissimilarity with all arrays and was interpreted as an outlier, as also confirmed by Principal Component Analysis (*PCA*; not shown).

Following batch correction for the 7 different acquiry dates, the arrays instead formed two distinct clusters for RA and OA in the hierarchical clustering dendrogram (Figure 1b). The effects of the different creation dates were completely removed and the outlier EB87 was integrated, as also confirmed by *PCA* (not shown). This unequivocal clustering of RA and OA patients was only achieved when correcting for 7 batches and not for 2 yearly batches only (2006 and 2009; Additional file 2: Figure S1).

Strikingly, distinct clustering for RA and OA following batch correction for the 7 different acquiry dates was not only observed at the 0 hour time point (i.e., before stimulation with TNF-α/TGF-β1), but also at all time points after stimulation except for the TGF-Î²1 late 12 hour time point (Additional file 3: Figure S2).

### Comparison of differentially expressed genes before and after batch correction

**Figure 2:**
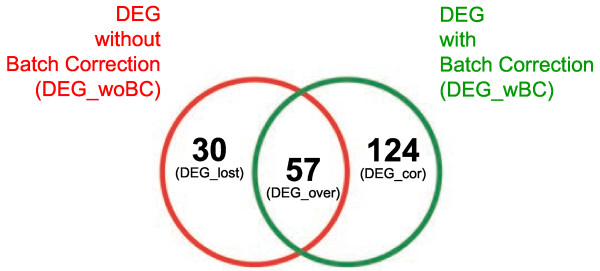
**Venn Plot for genes differentially expressed between RA and OA at time point 0 hours.** BC results in a doubling of differentially expressed genes (87 → 181). A total of 57 genes (65**.**51%) were represented in the intersection of the two gene lists (DEG_over).

*Limma* was used to obtain differentially expressed genes (DEG; ≥ 2-fold change; p-value of ≤ 0.05) for the key question concerning a generic difference between RA and OA patients. For the uncorrected data, 87 genes were extracted (DEG_woBC). The batch-corrected data contained 181 genes (DEG_wBC), with a total overlap of 65.51% (in total 57 genes; DEG_over) between the 2 gene sets. This was illustrated using a Venn plot (Figure 2).

For both data sets (DEG_woBC and DEG_wBC), a gene enrichment analysis was done with *GOstats* (p-value ≤ 0.05). In both data sets, the highest ranked GO term was “extracellular matrix structural constituent” with a p-value of 5.87E-03 and 3/74 genes for the uncorrected and a p-value of 7.60E-07 and 8/74 genes for the corrected data set (Table 3). In addition, 5 of 7 GO categories identified before batch correction were also overrepresented in the data set after batch correction, indicating that no major information was lost upon batch correction (Table 3). Furthermore, the p-value of the 3 genes from the top-ranked GO term contained in both data sets largely improved following batch correction (by up to 20 orders of magnitude), underlining the validity of the differential expression (Table 4).

**Table 3:** GO categories overrepresented in the gene set DEG_wBC (time point 0 hours) top-ranked with b) or without BC a)

**TermID**	**Members (DEG/all)**	**P-value**	**Category**
**a)**			
GO:0005201	3/74	5.87e-03	extracellular matrix structural constituent
GO:0050840	2/26	6.62e-03	extracellular matrix binding
GO:0005100	2/28	7.65e-02	Rho GTPase activator activity
GO:0008307	2/40	1.52e-02	structural constituent of muscle
GO:0005178	2/57	2.96e-02	integrin binding
GO:0001871	3/154	3.61e-02	pattern binding
GO:0030246	3/156	3.70e-02	carbohydrate binding
**b)**			
**GO:0005201**	**8/74**	**7.60E-07**	**extracellular matrix structural constituent**
GO:0048407	4/11	3.29E-06	platelet-derived growth factor binding
GO:0005540	3/19	9.04E-04	hyaluronic acid binding
GO:0015355	2/5	1.02E-03	secondary active monocarboxylate transmembrane transporter activity
GO:0004035	2/6	1.52E-03	alkaline phosphatase activity
**GO:0005178**	**4/57**	**2.74E-03**	**integrin binding**
GO:0004000	2/9	3.58E-03	adenosine deaminase activity
**GO:0050840**	**2/26**	**7.46E-03**	**extracellular matrix binding**
**GO:0008307**	**3/40**	**7.88E-03**	**structural constituent of muscle**
GO:0046332	3/42	9.02E-03	SMAD binding
GO:0030674	3/44	1.02E-02	protein binding
GO:0016853	4/83	1.02E-02	isomerase activity
GO:0000287	10/441	1.53E-02	magnesium ion binding
GO:0005507	3/62	2.56E-02	copper ion binding
GO:0042802	7/289	3.17E-02	identical protein binding
**GO:0005100**	**2/28**	**3.31E-02**	**Rho GTPase activator activity**
GO:0005518	2/31	3.94E-02	collagen binding
GO:0016903	2/33	4.47E-02	oxidoreductase activity

GO categories overrepresented in both data sets are highlighted in bold. Please note that both the number of the category members and the p-values for differential expression considerably improve by BC.

**Table 4:** Genes of the top-ranked GO category (time point 0 hours) identified with b) or without BC a)

**GC ID**	**P-value**	**SYMBOL**	**ENTREZ ID**	**Gene name**
GC01M103055_at	3.90E-05	COL11A1	1301	collagen, type XI, alpha 1
GC09P115957_at	3.73E-03	COL27A1	85301	collagen, type XXVII, alpha 1
GC0XP152413_at	3.59E-02	BGN	633	biglycan
**GC01M103055_at**	**5.31E-25**	**COL11A1**	**1301**	**collagen, type XI, alpha 1**
**GC0XP152413_at**	**5.07E-12**	**BGN**	**633**	**biglycan**
GC02P189547_at	1.39E-08	COL3A1	1281	collagen,type III, alpha 1
**GC09P115957_at**	**2.98E-06**	**COL27A1**	**85301**	**collagen, type XXVII, alpha 1**
GC01P031814_at	6.96E-05	TINAGL1	64129	tubulointerstitial nephritis antigen-like 1
GC07P093861_at	4.37E-04	COL1A2	1278	collagen, type I, alpha 2
GC02M189604_at	1.13E-03	COL5A2	1290	collagen, type V, alpha 2
GC17M045617_at	1.28E-02	COL1A1	1277	collagen, type I, alpha 1

Genes of the GO category (GO:0005201 extracellular matrix structural constituent) top-ranked with or without BC (see Table 3), including their p-value for differential expression between RA and OA. Genes represented in both data sets are highlighted in bold. Please note that both the number of genes and the p-value for differential expression considerably improve.

**Figure 3:**
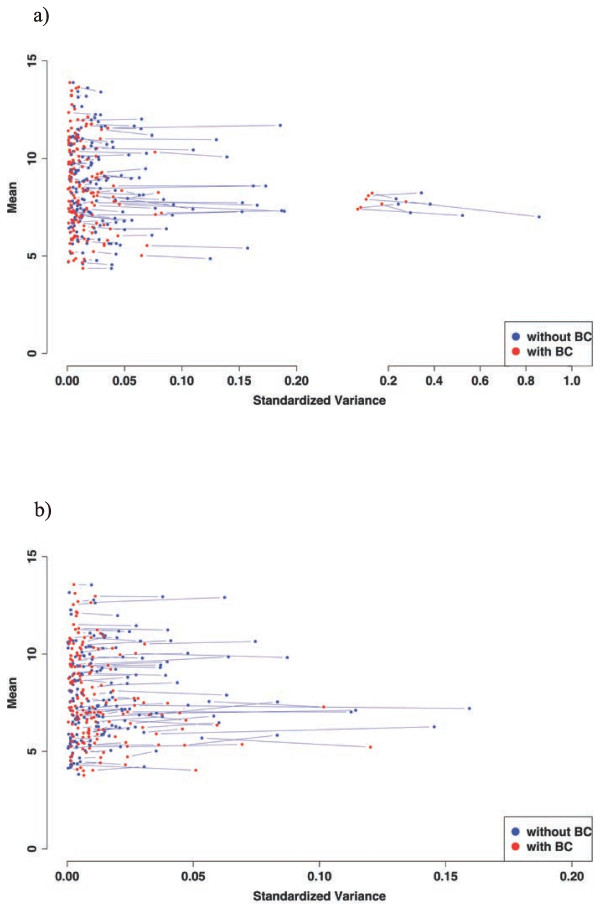
**Means and variances of 181 differentially expressed genes from the DEG_wBC set (time point 0 hours) in RA (a; n = 12; i.e. 6 patients with two replicates each) and OA (b; n = 12; i.e. 6 patients with two replicates each) with (red dots) or without BC (blue dots). **There are generally only marginal changes of the means, but moderate to substantial reductions of the variances, as indicated by an exclusively horizontal shift.

To analyze the magnitude of changes in either mean or standardized variance induced by the batch correction, variance/mean plots were generated to illustrate the “shrinkage” of the expression values. For this purpose, the variances were standardized (range between 0 and 1) to prevent negative values. Analyzing the 181 genes from the DEG_wBC data set differentially expressed between RA and OA, only marginal changes of the means, but moderate to substantial reductions of the variances were observed for all genes (Figure 3).

**Figure 4:**
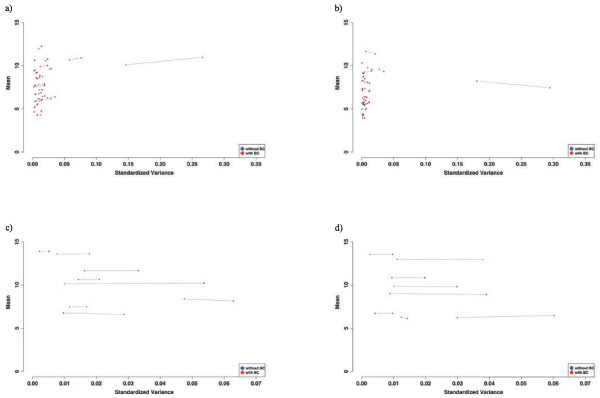
**Means and variances of differentially expressed genes from the DEG_lost set (a,b; for definition see Figure**2**) and the DEG_interest set (c,d; for definition see Figure**2**) in RA (a, c; n = 12) and OA (b, d; n = 12) patients with (red dots) or without BC (blue dots). **There are generally only marginal changes of the means, but moderate to substantial reductions of the variances.

The same was true when the genes lost for analysis following batch correction (DEG_lost; Figure 4a,b), the genes of interest from the GO term “extracellular matrix structural constituent” (DEG_interest; Figure 4c,d) or all genes identified without batch correction (DEG_woBC; Additional file 4: Figure S3) were displayed in the variance/mean plot.

To further analyze the magnitude of changes in the mean induced by the batch correction, the mean values in both data sets were compared and the changes expressed as percentages of the initial value; in both RA and OA, very limited changes were observed (maximal change 4.087%), either at the time point 0 hours (Table 5) or at any time point following stimulation with TNF-α or TGF-β1 (Additional file 1: Table S1). Despite the limited changes of the means induced by BC, BC resulted in a substantial improvement of the power for the differentiation of RA and OA for all genes of interest (Additional file 5: Table S2).

**Table 5:** Mean expression values of differentially expressed genes (time point 0 hours)

**Symbol**	**GCID**	**Without BC**	**With BC**	**Change (%)**	**Without BC**	**With BC**	**Change (%)**
COL1A1	GC17M045617_at	10.663	10.636	-0.25	9.793	9.852	+0.60
COL1A2	GC07P093861_at	13.894	13.895	+0.01	13.544	13.562	+0.13
COL3A1	GC02P189547_at	13.616	13.599	-0.12	12.937	12.967	+0.23
COL5A2	GC02M189604_at	11.664	11.659	-0.04	10.841	10.852	+0.10
COL11A1	GC01M103055_at	8.141	8.373	+2.84	6.470	6.230	-3.70
BGN	GC0XP152413_at	10.201	10.131	-0.68	8.912	8.995	+0.93
TINAGL1	GC01P031814_at	6.580	6.742	+2.46	6.277	6.125	-2.42
COL27A1	GC09P115957_at	7.454	7.463	+0.12	6.688	6.703	+0.23

Mean expression values of differentially expressed genes of interest in RA and OA patients with and without BC (time point 0 hours); there are only very limited changes of the means in either RA or OA (up to 3.7%). For the full data of all stimuli and time points see Additional file 1: Table S1.

There was no indication for any type-1 error concerning differential expression of the genes of interest, as demonstrated by permuting the disease status (RA and OA) 20 times with subsequent BC; applying the thresholds 2-FC and p < 0.05, none of the 8 genes of interest were contained in the list of differentially expressed genes for any of the permutations (Additional file 6: Table S3). Also, a complete lack of clustering for RA and OA was observed and, strikingly, the pairs of one patient for the time point 0 were separated in some cases (Additional file 7: Figure S4)

**Figure 5:**
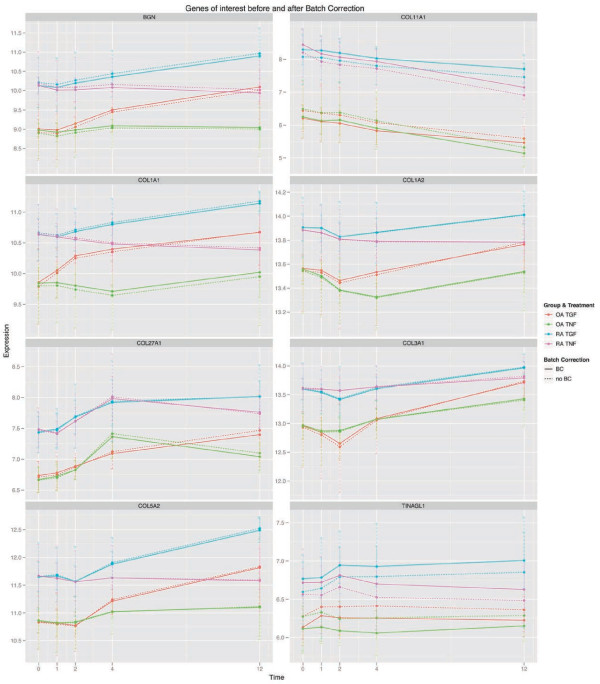
**Time courses of genes of interest (DEG_interest; see Table**4**b) in synovial fibroblasts from RA patients (blue and purple) or OA (red and green) stimulated with TNF-α (red and blue) or TGF-β1 (green and purple).** There were only marginal differences for the gene expression values with or without BC (see also Additional file 6: Table S1). As expected, there was a clearly different regulation of the expression of 6 of 8 genes (BGN, COL1A1, COL27A1, COL5A2, COL1A2 and COL3A1; for definition of the abbreviations see Table 4) following stimulation of with either TNF-α or TGF-β1; this differential regulation was common for SFB from RA and OA patients (see Additional file 6: Table S1). However, 2 of 8 genes (COL11A1 and TINAGL) were regulated in a similar fashion by TNF-α and TGF-β1 in both RA and OA patients. Strikingly, significant differences between RA and OA patients were observed for all genes of interest already at the time point 0 hours (see Additional file 8: Table S4a). These differences were unaffected by stimulation with either TNF-α or TGF-β1.

In addition to the differential expression between RA and OA after BC for the 8 genes of interest from the top-ranked GO term at time point 0 hours (Table 4b), these genes also remained differentially expressed between RA and OA at all time points following stimulation with either TNF-α or TGF-β1 (Figure 5, Additional file 8: Table S4).

## Discussion

In the present study, the *ComBat* method was highly effective in removing batch effects due to different acquiry dates of the microarrays; this was demonstrated by i) unequivocal clustering of the RA and OA patients in the batch-corrected data for almost all time points investigated (shown by hierarchical clustering dendrograms and *PCA*); ii) integration of the outlier EB87, resulting in reduced standardized variances for a number of genes; iii) identification of a large number of genes differentially expressed between RA and OA with highly significant p-values (up to 5.31E-25); iv) identification of numerous overrepresented GO terms (with an increased number of members and strongly improved p-values).

The 7 different batches (aquiry dates) were clearly grouped into the two main clusters representing microarray analyses from the years 2006 and 2009. These 2 main clusters are a clear example of inevitable batch effects since the supplier of the hybridization agents changed the chemical components used in the production process in the year 2007 (personal communication; Affymetrix). The only possibility to avoid such batch effects would have been to collect all the samples for simultaneous analysis, an approach technically impossible for a total of 120 samples at the time point of analysis (and possibly even today). In addition, unequivocal clustering of RA and OA patients was only achieved when correcting for 7 batches and not only for 2 batches (2006 and 2009), indicating that both the technical change of the supplier and a basic variance among the acquiry dates contributed to the dissimilarity of the arrays. This should be taken into consideration for future microarray analyses.

The batch correction had no major influence on the underlying data, as demonstrated by i) almost unaffected means (maximal change 4.087%) in the variance/mean plots for a number of different gene sets; ii) marginally changed, but mostly reduced standardized variances for the same gene sets; iii) substantial overlap (> 65%) of the genes differentially expressed between RA and OA at time point 0 hours before and after batch correction; iv) preservation of 5/7 of the top-ranked GO categories after batch correction. These findings show that *ComBat* is highly suitable for batch correction of data derived from small sample sizes and does not lead to inappropriate modification of the underlying data as previously published [2].

The genes up-regulated at time point 0 hours (identified following batch correction) reflected a key feature of RA, i.e., SFB-driven fibrosis of the affected joints [26]. In particular, the enhanced expression of collagens type I and III (COL1A1, COL1A2, COL3A1), as well as biglycan (BGN) by RA-SFB has been previously published [27-29]. In the context of RA, collagens types V and XI are less well known as mediators of fibrosis, but are targets of matrix metalloproteinase-mediated proteolytic activity [30,31]. However, together with collagens type I, II, and III, these collagens are defined as (fibrillar) interstitial collagens [32] and represent major components of the extracellular matrix [33], thus suggesting a potential role in fibrosis also for these proteins. The fibrillar type XXVII collagen (COL27A1) and lipocalin-7 (also known as tubulointerstitial nephritis antigen-like 1; TINAGL1), which were identified as up-regulated molecules in RA-SFB in this study for the first time, are not intrinsic extracellular matrix molecules, but both exhibit differentiation potential. Type XXVII collagen, for example, is predominantly expressed in cartilaginous tissues and generally involved in skeletogenesis [34], whereas matricellular lipocalin-7 appears to be a (positive) regulator of angiogenesis [35], potentially influencing enhanced angiogenesis in the synovial membrane [36]. Taken together, the enhanced formation of these matrix components may contribute to joint fibrosis in an attempt to counteract the progressive destruction of cartilage and bone.

Strikingly, differential expression of the 8 genes of interest from the top-ranked GO term “extracellular matrix structural constituent” (DEG_interest) was not only observed at the time point 0 hours, but also at all time points following stimulation with either TNF-α or TGF-β1. This suggests that RA-SFB show a constitutively altered “rheumatic phenotype”, which is preserved upon stimulation with TNF-α and TGF-β1 (“spacer effect”). Possible reasons for such an “imprinted” RA phenotype may include both genomic changes, e.g., mutations or chromosome aberrations, or epigenetic modifications, e.g. methylation or histone acetylation status [37-39].

## Conclusion

The present study clearly underscores the necessity of correction and removal of batch effects in the analysis of microarray data. The application of batch correction allowed the unequivocal identification of genes differentially expressed between RA and OA and returned the top-ranked GO category “extracellular matrix structural constituent” (8/74 genes; p-value decreased by 4 orders of magnitude). Batch correction strongly reduced the variance, but only marginally influenced the mean expression levels, i.e., led to reliable results without falsification of the underlying data.

RA-SFB show a constitutively altered “rheumatic phenotype”, which is preserved upon stimulation with TNF-α and TGF-β1, suggesting an “imprinted” RA phenotype.

## Abbreviations

ACR: American College of Rheumatology; CDF: Chip Definition File; ComBat: Combating Batch Effects When Combining Batches of Gene Expression Microarray Data; DEG: Differentially expressed genes; DMEM, Dulbecco's modified Eagle's medium; EB: Empirical Bayes; GO: Gene Ontology; hg: Hypergeometric; L/S: Location and scale; OA: Osteoarthritis; PCA: Principal Component Analysis; RA: Rheumatoid arthritis; RMA: Robust Multi-Array Analysis; SFB: Synovial fibroblasts.

## Competing interests

The authors declare that they have no competing interests.

## Authors’ contributions

PK performed the bioinformatic analysis, contributed to the design of the study, and participated in the layout, writing, and finalization of the manuscript. RG and RWK contributed to the design of the study and participated in the layout, writing, and the finalization of the manuscript. DP, RH, and DK performed the experiments with synovial fibroblasts and the respective data analysis and participated in writing the manuscript. All authors read and approved the final manuscript.

## Pre-publication history

The pre-publication history for this paper can be accessed here:

http://www.biomedcentral.com/1755-8794/5/23/prepub

## Supplementary Material

Additional file 1 **Table S1.** Mean expression values of differentially expressed genes of interest in RA and OA patients with and without BC for all time points. Click here for file

Additional file 2 **Figure S1.** Hierarchical clustering of uncorrected and batch-corrected data from time point 0: a) The uncorrected data form clusters reflecting the 2 different years of acquiry (red shades for arrays generated in 2006; blue shades for those generated in 2009). In contrast, RA and OA are not grouped. b) The *ComBat*-corrected data (2 batches) still fail to form clusters reflecting the diseases (RA and OA). Click here for file

Additional file 3 **Figure S2.** Cluster plots for the time points 1, 2, 4, and 12. a) Time point 1: TNF-α. b) Time point 1: TGF-β1. c) Time point 2: TNF-α. d) Time point 2: TGF-β1. e) Time point 4: TNF-α. f) Time point 4: TGF-β1. g) Time point 12: TNF-α. h) Time point 12: TGF-β1. Click here for file

Additional file 4 **Figure S3.** Means and variances of differentially expressed genes from the uncorrected data set (DEG_woBC) in RA (a) and OA (b) patients with (red dots) or without BC (blue dots); there are generally only marginal changes of the means, but moderate to substantial reductions of the variances. Click here for file

Additional file 5 **Table S2.** Comparison of the power values for the differentiation of RA and OA before and after BC for the differentially expressed genes [applied values: means ± standard deviations (SD)] calculated using GPower [25]. Click here for file

Additional file 6 **Table S3.** Differentially expressed genes calculated with LIMMA for 20 permutations. Click here for file

Additional file 7 **Figure S4.** Cluster plots for time point 0 on the basis of 20 permutations of the disease status (RA and OA). Click here for file

Additional file 8 **Table S4.** Mann–Whitney U tests for the comparison between RA and OA in a), or for the comparison between TNF-α and TGF-β1 in b); +, significant difference; -, lack of significant difference; *, significance test is not applicable (technical replicates). Click here for file
