# Supplementary figures and images for: Batch correction of microarray data substantially improves the identification of genes differentially expressed in Rheumatoid Arthritis and Osteoarthritis
Source: BMC Med Genomics. 2012 Jun 8;5:23. doi: 10.1186/1755-8794-5-23 (PMC3528008; doi:10.1186/1755-8794-5-23)

a)

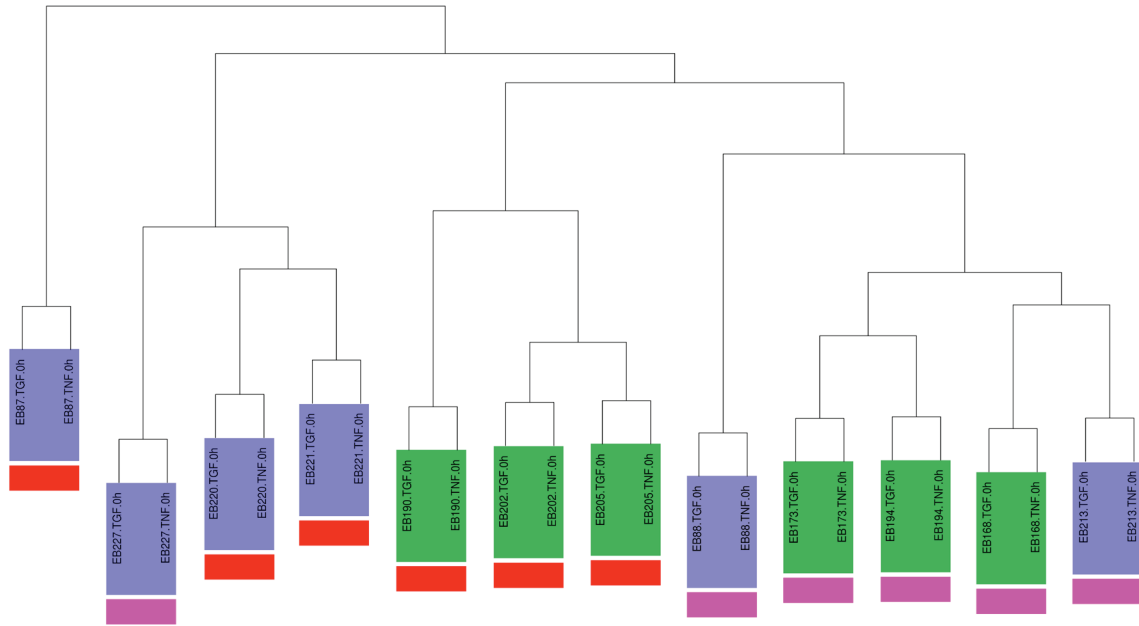

b)

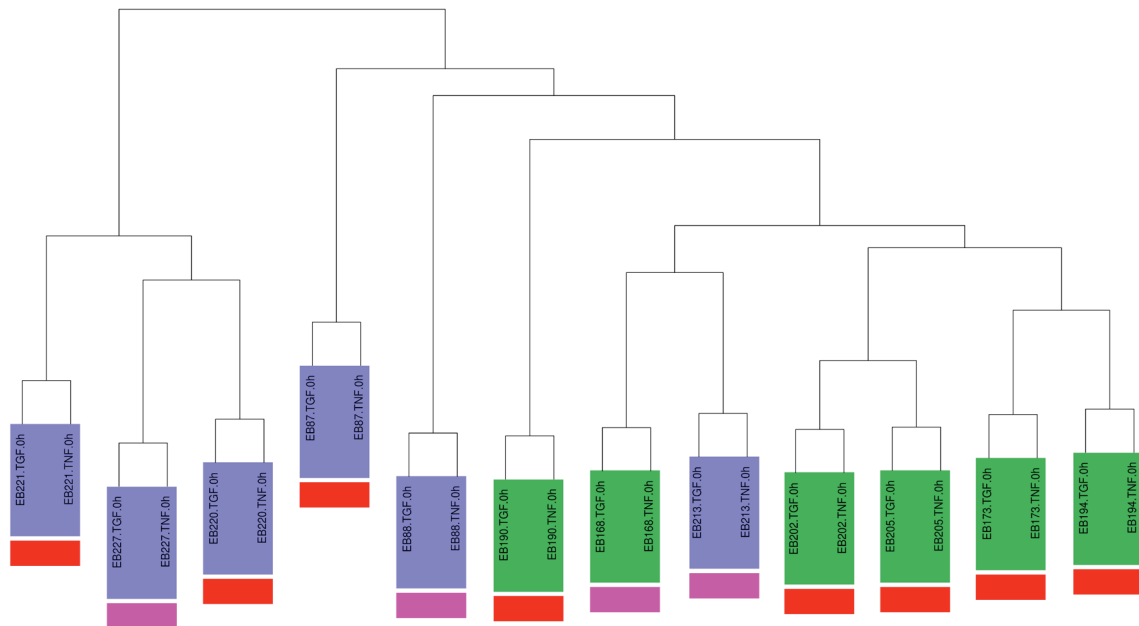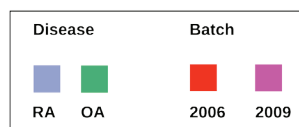

Supplement: Additional file 2 — Figure S1. Hierarchical clustering of uncorrected and batch-corrected data from time point 0: a) The uncorrected data form clusters reflecting the 2 different years of acquiry (red shades for arrays generated in 2006; blue shades for those generated in 2009). In contrast, RA and OA are not grouped. b) The ComBat-corrected data (2 batches) still fail to form clusters reflecting the diseases (RA and OA). [file 1755-8794-5-23-S2.pdf]

a)

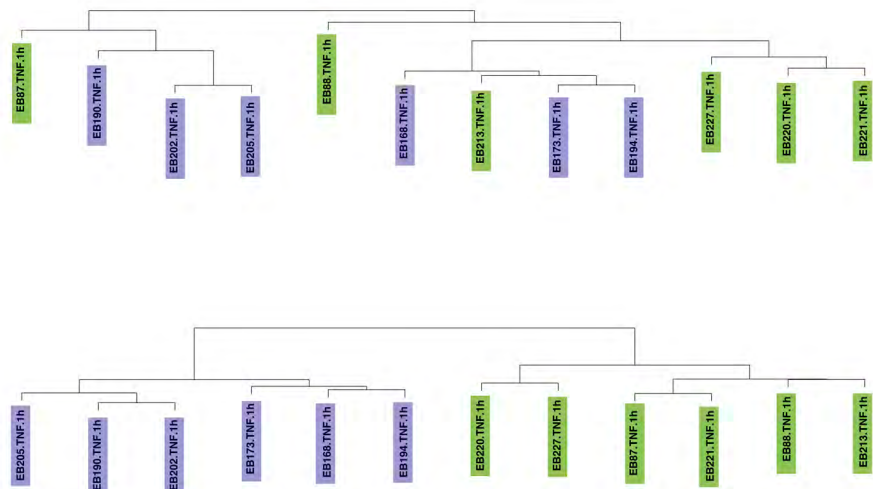

b)

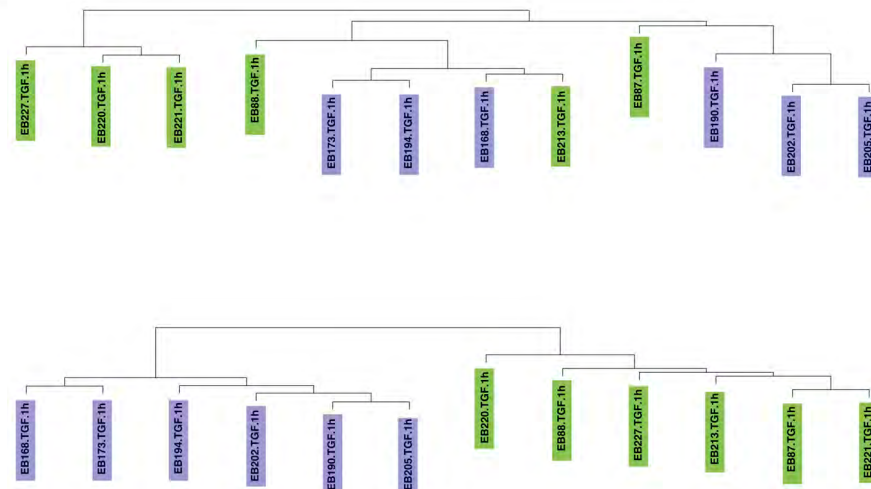

c)

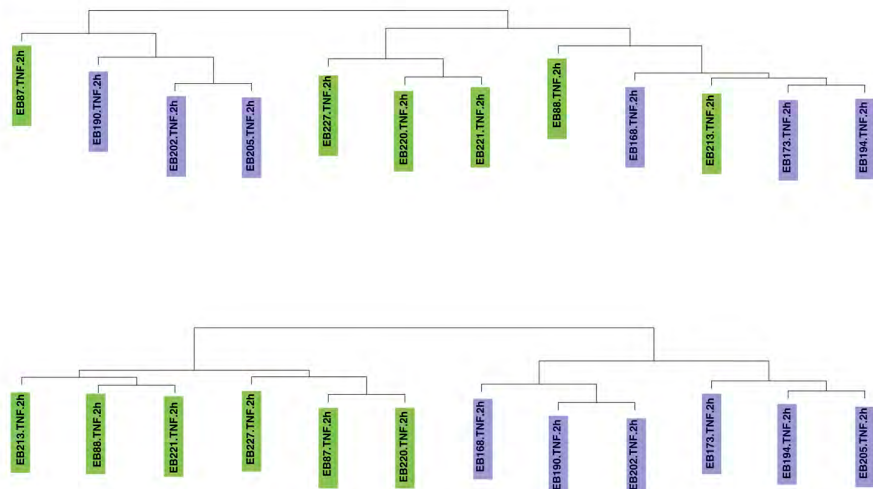

d)

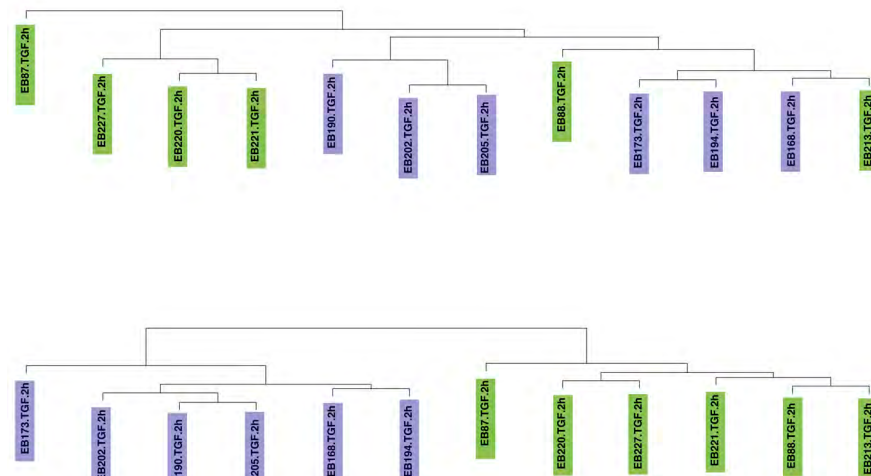

e)

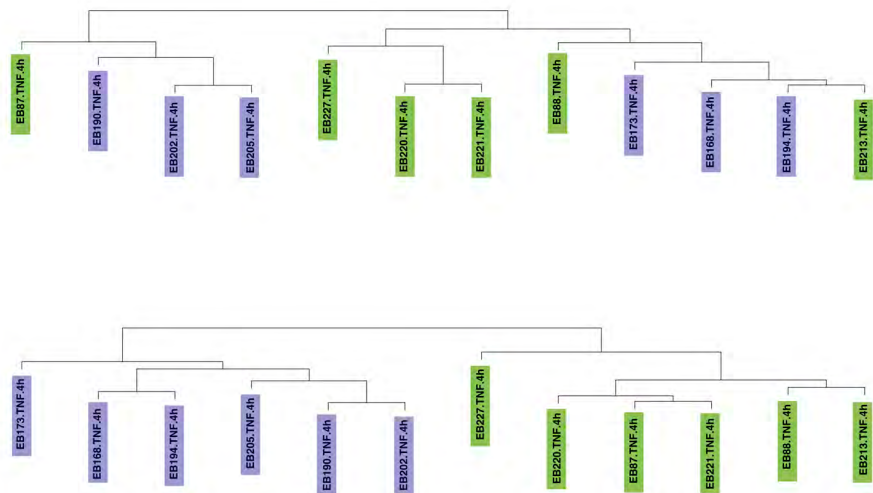

f)

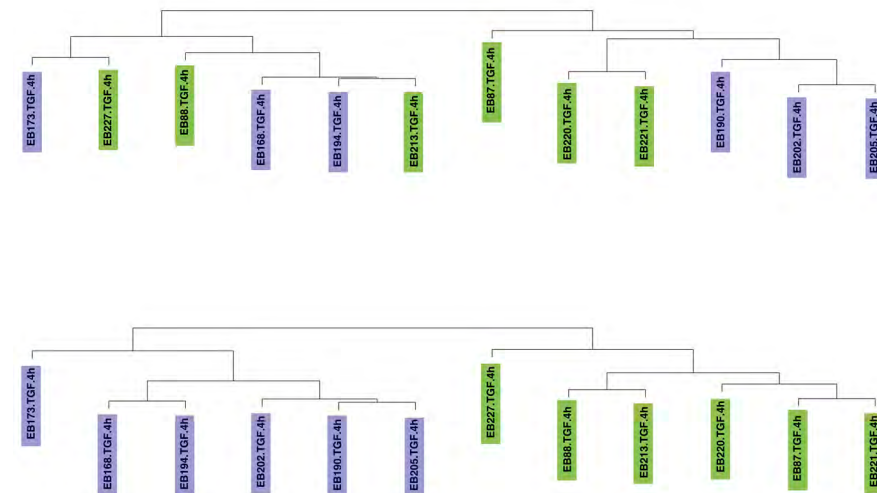

g)

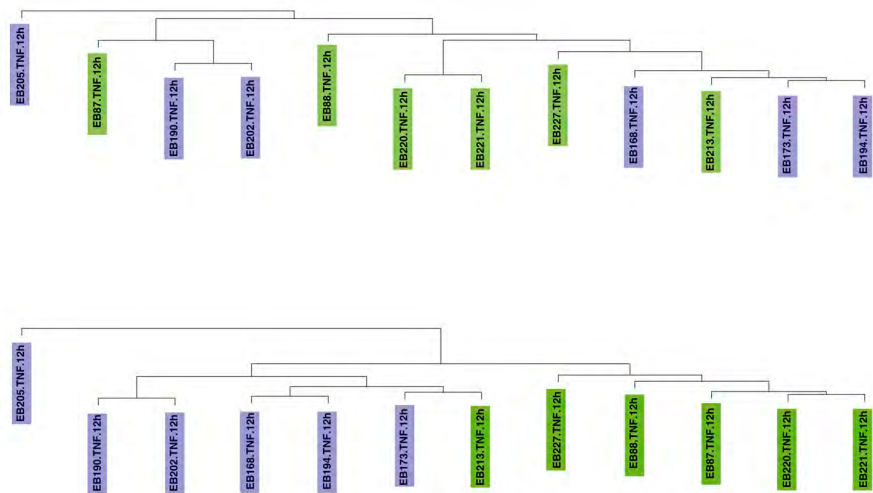

h)

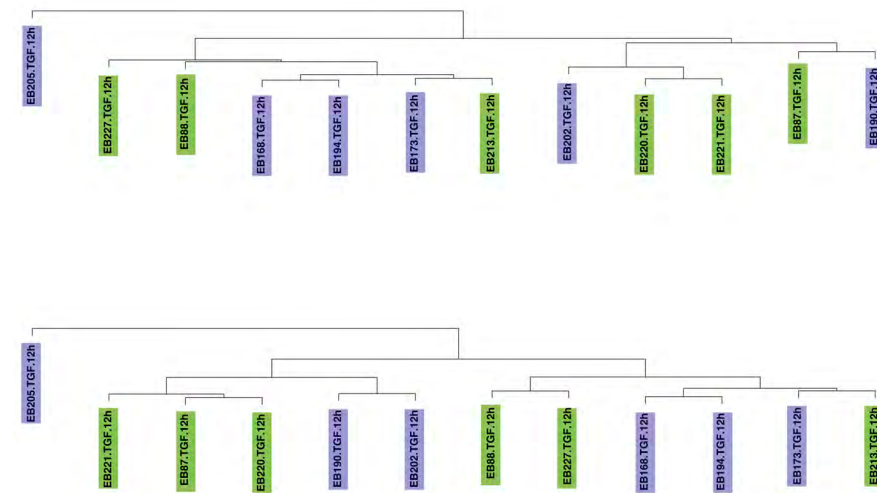

Supplement: Additional file 3 — Figure S2. Cluster plots for the time points 1, 2, 4, and 12. a) Time point 1: TNF-α. b) Time point 1: TGF-β1. c) Time point 2: TNF-α. d) Time point 2: TGF-β1. e) Time point 4: TNF-α. f) Time point 4: TGF-β1. g) Time point 12: TNF-α. h) Time point 12: TGF-β1. [file 1755-8794-5-23-S3.pdf]

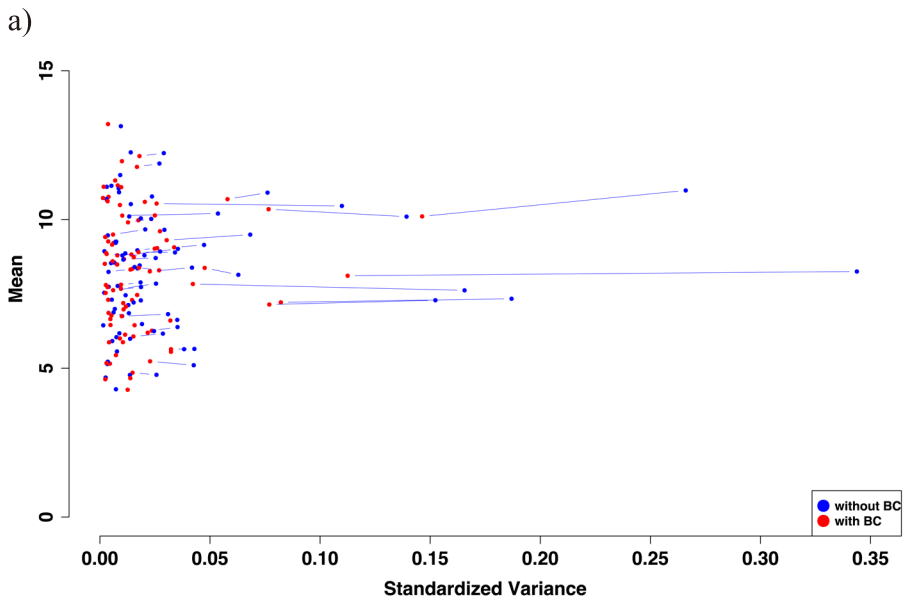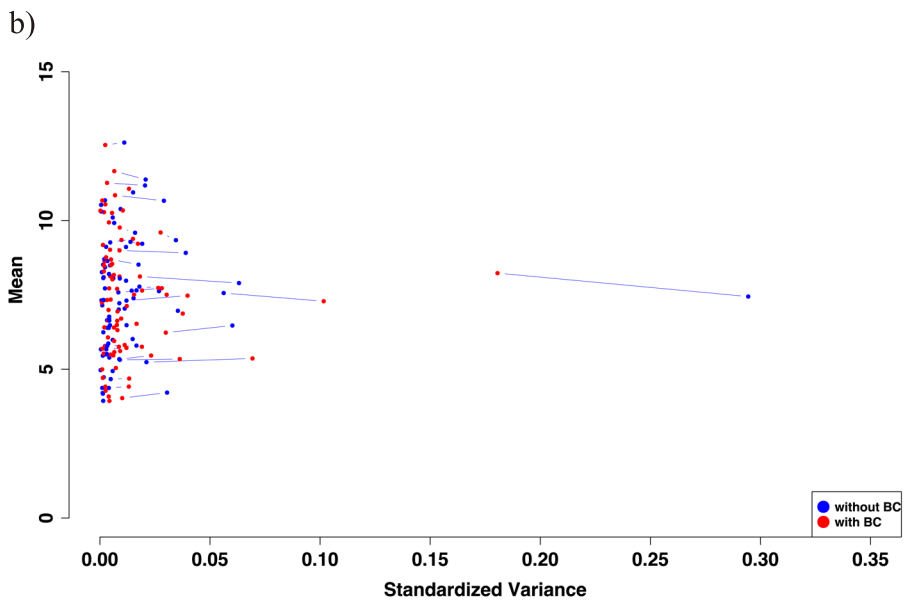

Supplement: Additional file 4 — Figure S3. Means and variances of differentially expressed genes from the uncorrected data set (DEG_woBC) in RA (a) and OA (b) patients with (red dots) or without BC (blue dots); there are generally only marginal changes of the means, but moderate to substantial reductions of the variances. [file 1755-8794-5-23-S4.pdf]
