# Supplementary material for: Batch correction of microarray data substantially improves the identification of genes differentially expressed in Rheumatoid Arthritis and Osteoarthritis
Source: BMC Med Genomics. 2012 Jun 8;5:23. doi: 10.1186/1755-8794-5-23 (PMC3528008; doi:10.1186/1755-8794-5-23)

t0: Complete Cluster with Batch Correction and 2 groups (RA/OA), Permutation=1

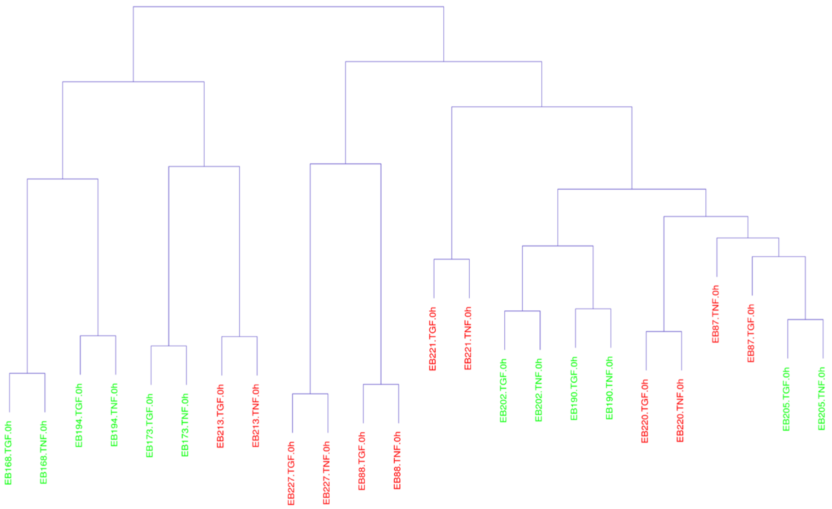

t0: Complete Cluster with Batch Correction and 2 groups (RA/OA), Permutation=2

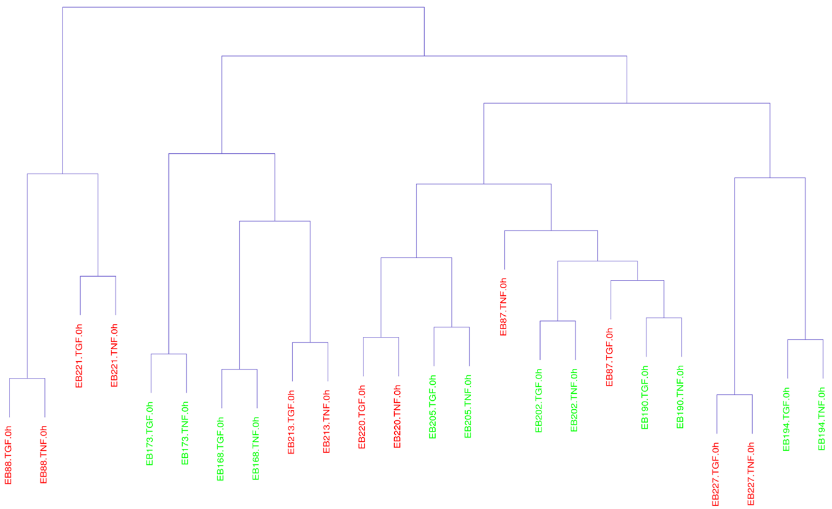

RA OA

t0: Complete Cluster with Batch Correction and 2 groups (RA/OA), Permutation=3

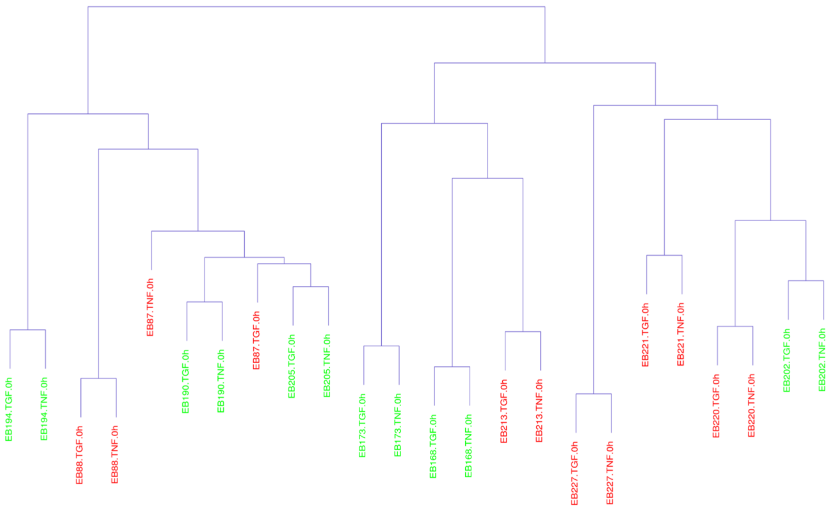

t0: Complete Cluster with Batch Correction and 2 groups (RA/OA), Permutation=4

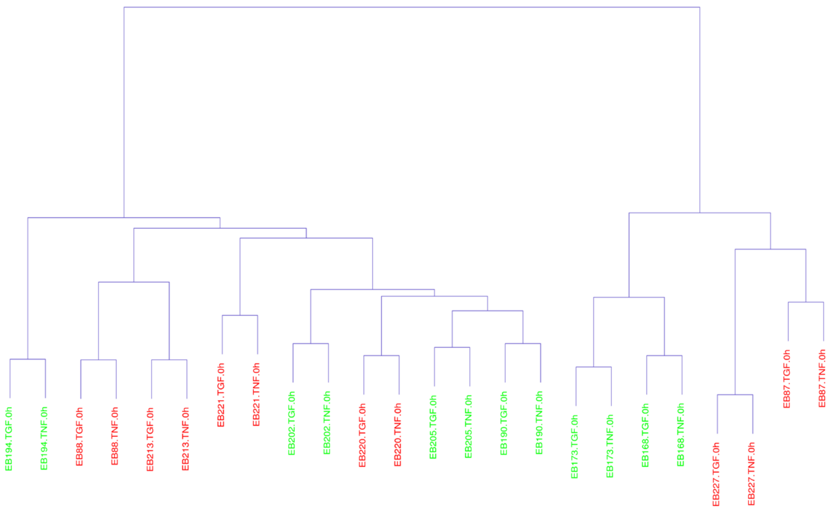

t0: Complete Cluster with Batch Correction and 2 groups (RA/OA), Permutation=5

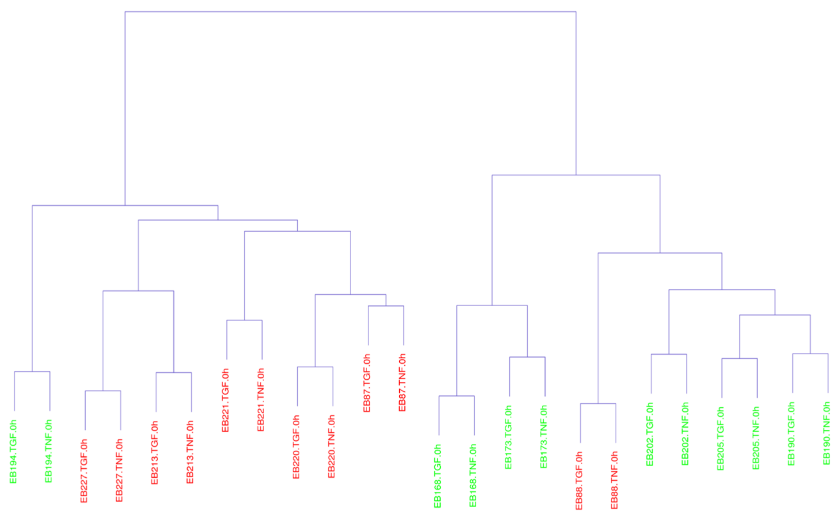

t0: Complete Cluster with Batch Correction and 2 groups (RA/OA), Permutation=7

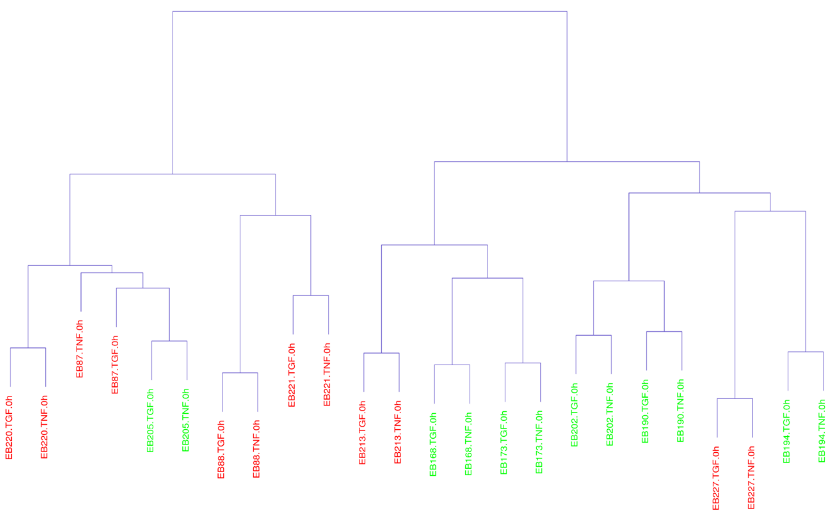

t0: Complete Cluster with Batch Correction and 2 groups (RA/OA), Permutation=6

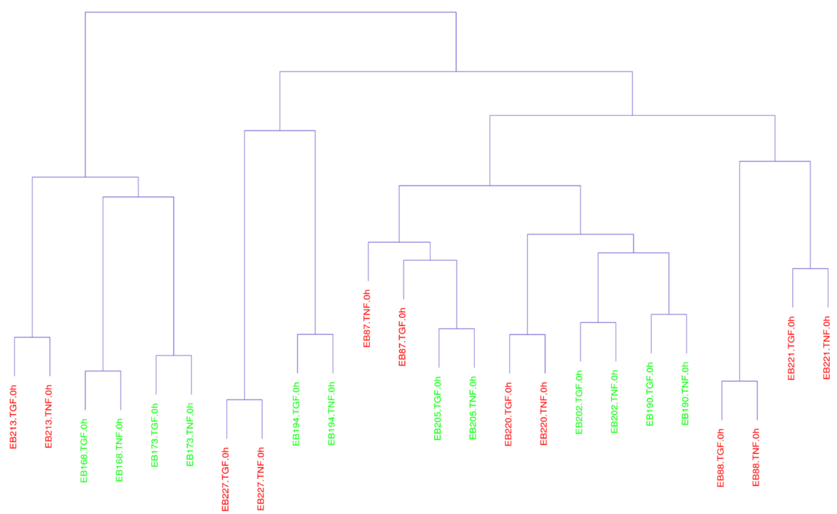

t0: Complete Cluster with Batch Correction and 2 groups (RA/OA), Permutation=8

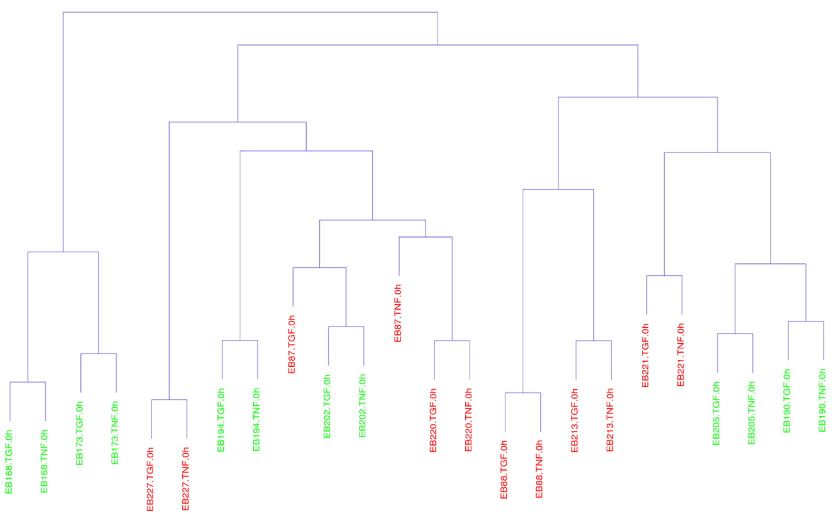

RA OA

t0: Complete Cluster with Batch Correction and 2 groups (RA/OA), Permutation=9

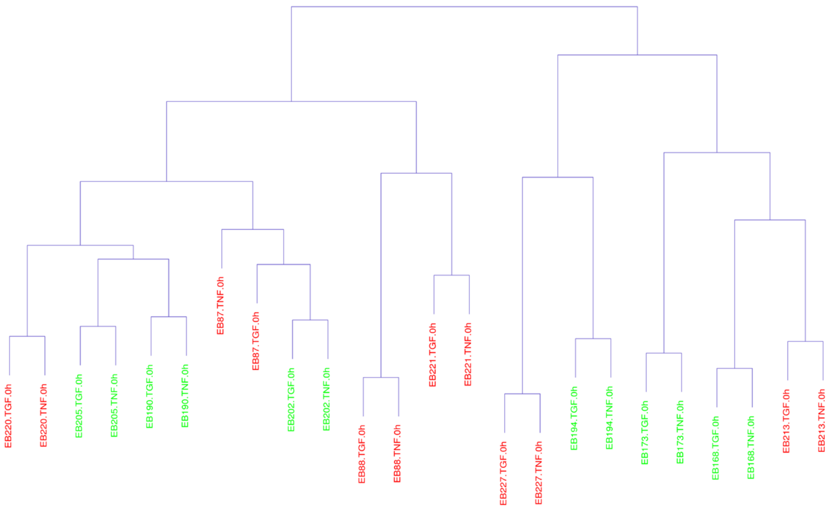

t0: Complete Cluster with Batch Correction and 2 groups (RA/OA), Permutation=11

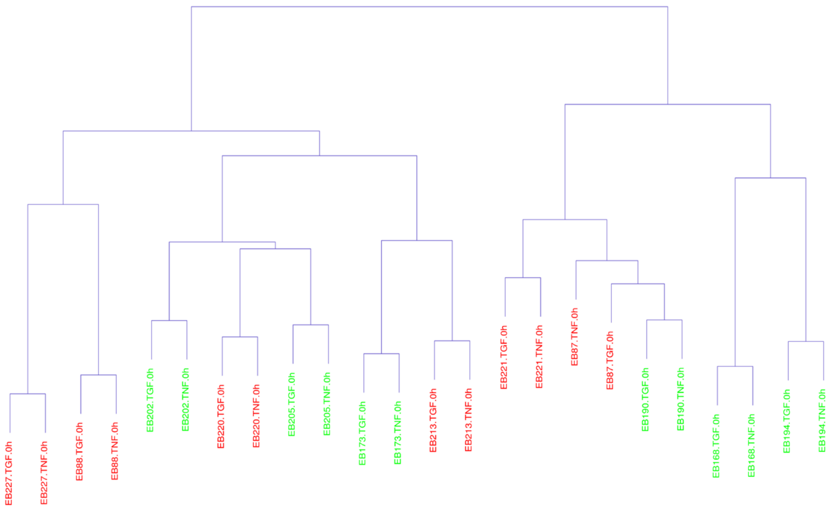

t0: Complete Cluster with Batch Correction and 2 groups (RA/OA), Permutation=10

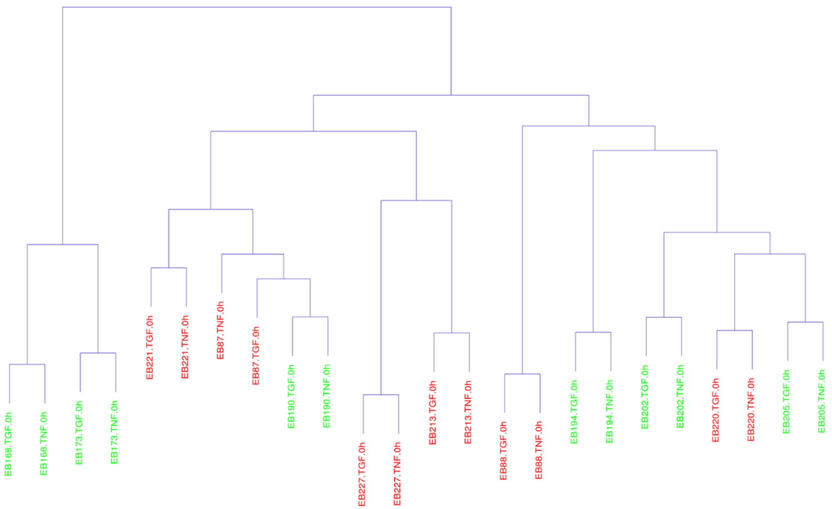

t0: Complete Cluster with Batch Correction and 2 groups (RA/OA), Permutation=12

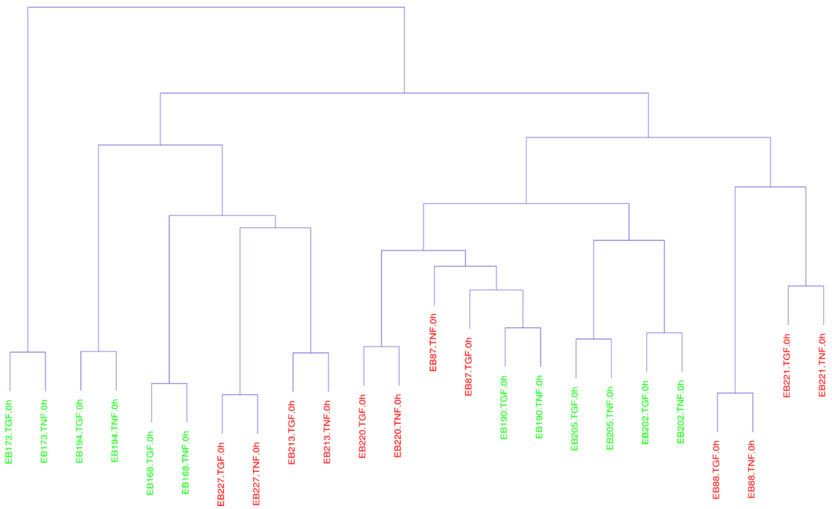

RA

OA

t0: Complete Cluster with Batch Correction and 2 groups (RA/OA), Permutation=13

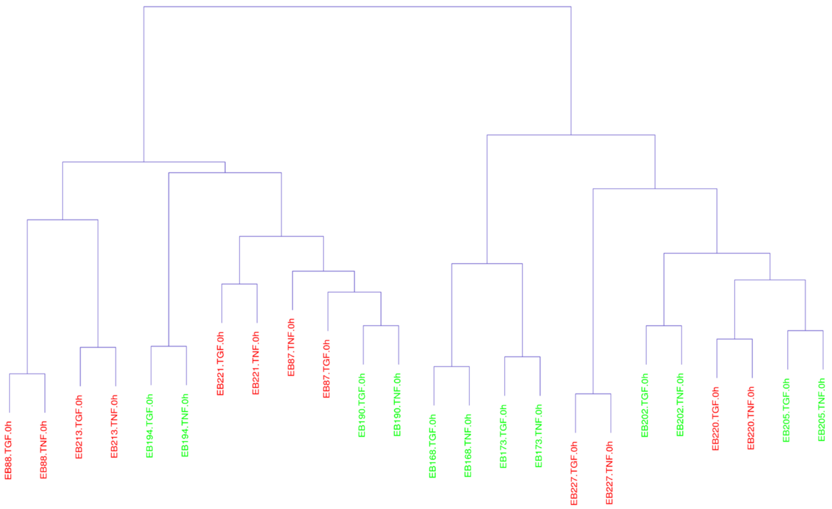

t0: Complete Cluster with Batch Correction and 2 groups (RA/OA), Permutation=15

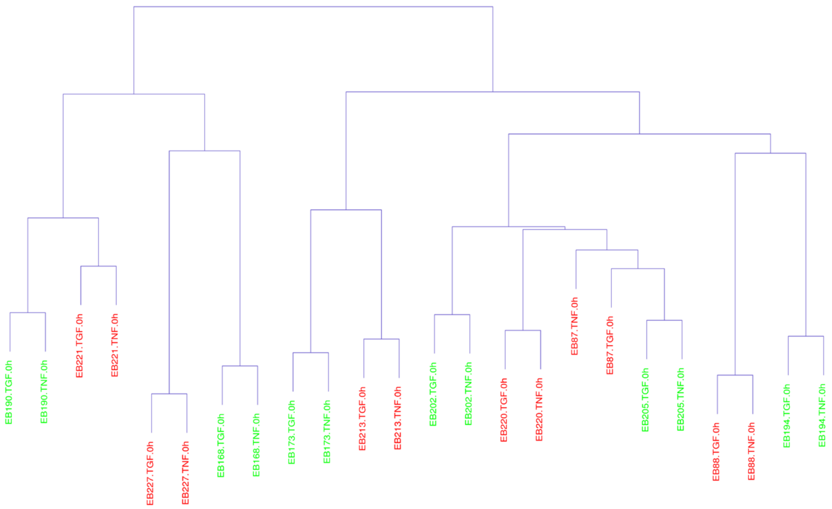

t0: Complete Cluster with Batch Correction and 2 groups (RA/OA), Permutation=14

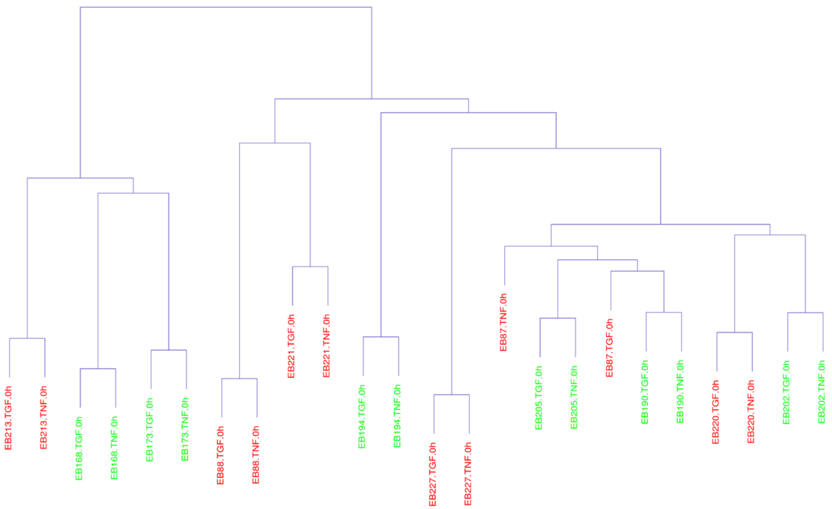

t0: Complete Cluster with Batch Correction and 2 groups (RA/OA), Permutation=16

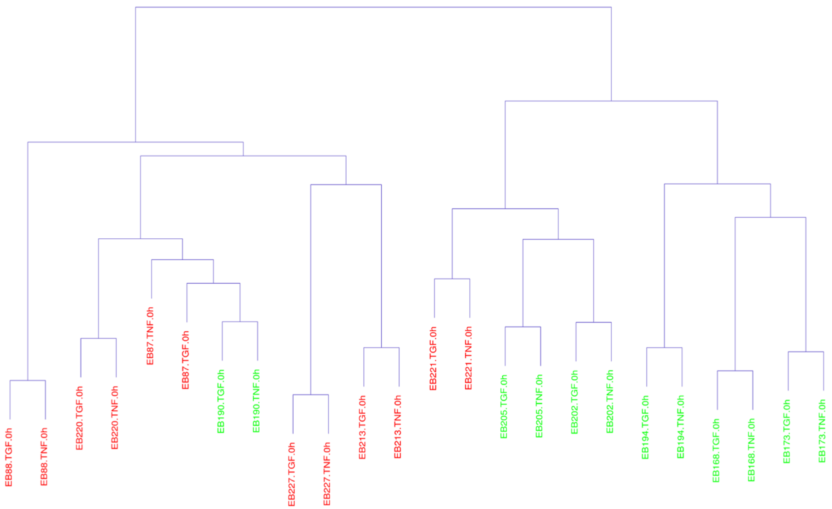

RA OA

t0: Complete Cluster with Batch Correction and 2 groups (RA/OA), Permutation=17

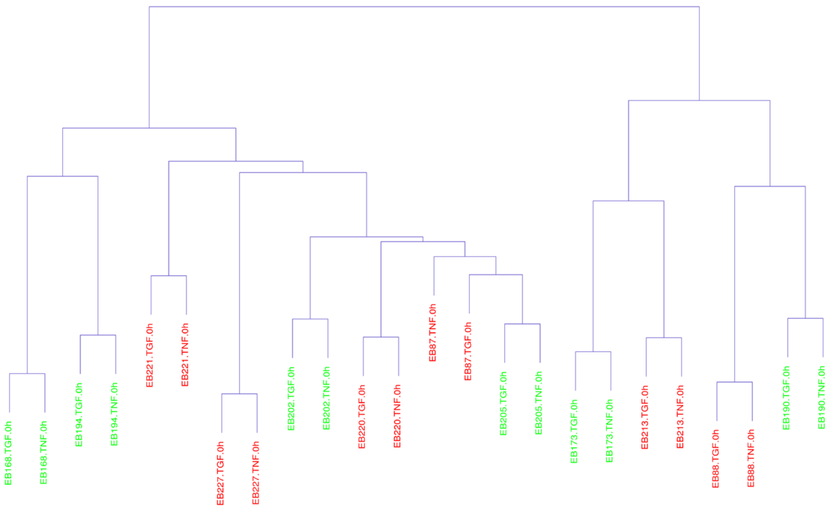

t0: Complete Cluster with Batch Correction and 2 groups (RA/OA), Permutation=19

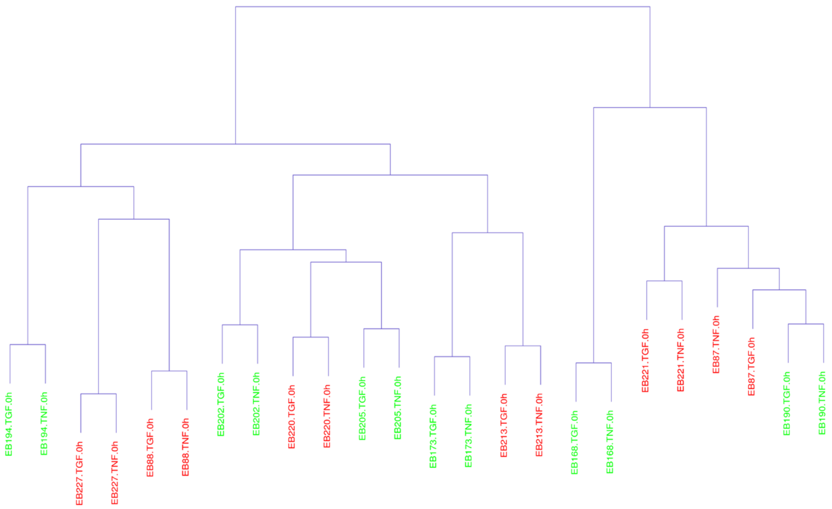

t0: Complete Cluster with Batch Correction and 2 groups (RA/OA), Permutation=18

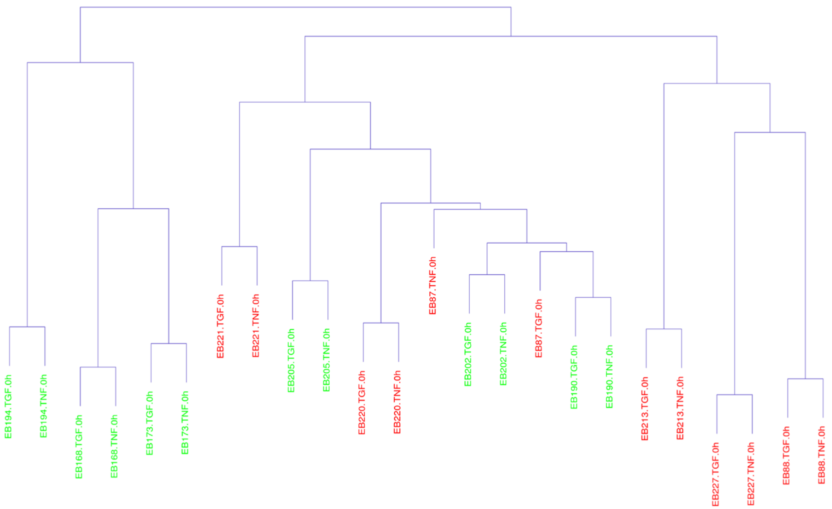

t0: Complete Cluster with Batch Correction and 2 groups (RA/OA), Permutation=20

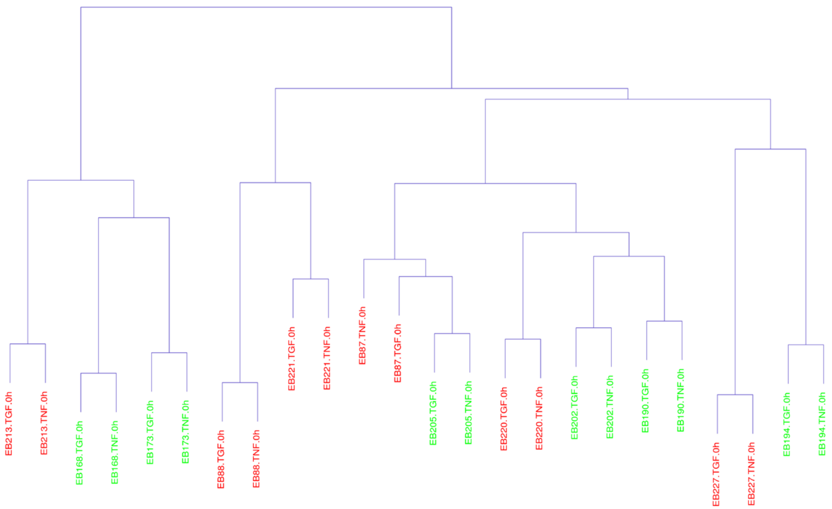

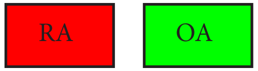

Supplement: Additional file 7 — Figure S4. Cluster plots for time point 0 on the basis of 20 permutations of the disease status (RA and OA). [file 1755-8794-5-23-S7.pdf]
